# Engineered Nanomaterials in Soil: Their Impact on Soil Microbiome and Plant Health

**DOI:** 10.3390/plants11010109

**Published:** 2021-12-30

**Authors:** Shams Tabrez Khan, Syed Farooq Adil, Mohammed Rafi Shaik, Hamad Z. Alkhathlan, Merajuddin Khan, Mujeeb Khan

**Affiliations:** 1 Department of Agricultural Microbiology, Faculty of Agricultural Sciences, Aligarh Muslim University, Aligarh 2002002, UP, India; 2 Department of Chemistry, College of Science, King Saud University, P.O. Box 2455, Riyadh 11451, Saudi Arabia; sfadil@ksu.edu.sa (S.F.A.); mrshaik@ksu.edu.sa (M.R.S.); khathlan@ksu.edu.sa (H.Z.A.); mkhan3@ksu.edu.sa (M.K.)

**Keywords:** engineered nanomaterials, ENMs in soil, soil and plant microbiome, soil and plant health

## Abstract

A staggering number of nanomaterials-based products are being engineered and produced commercially. Many of these engineered nanomaterials (ENMs) are finally disposed into the soil through various routes in enormous quantities. Nanomaterials are also being specially tailored for their use in agriculture as nano-fertilizers, nano-pesticides, and nano-based biosensors, which is leading to their accumulation in the soil. The presence of ENMs considerably affects the soil microbiome, including the abundance and diversity of microbes. In addition, they also influence crucial microbial processes, such as nitrogen fixation, mineralization, and plant growth promoting activities. ENMs conduct in soil is typically dependent on various properties of ENMs and soil. Among nanoparticles, silver and zinc oxide have been extensively prepared and studied owing to their excellent industrial properties and well-known antimicrobial activities. Therefore, at this stage, it is imperative to understand how these ENMs influence the soil microbiome and related processes. These investigations will provide necessary information to regulate the applications of ENMs for sustainable agriculture and may help in increasing agrarian production. Therefore, this review discusses several such issues.

## 1. Introduction

Nanotechnology has become an integral part of developing societies due to its ever-growing applications in diverse fields [1,2]. The growth of the nano-based industry mainly relies on the fabrication, manipulation, and deployment of advanced nanomaterials for numerous applications to solve current and future challenges faced by various sectors [3,4]. According to different estimates, thousands of nanomaterials-based commercial products are currently available in the market, which is expected to increase further in the future [5]. Nanomaterials, which are intentionally manufactured, unlike naturally occurring nanomaterials such as montmorillonite (MMT, kaolinite, saponite, etc.) are typically referred to as engineered nanomaterials (ENMs). Nanomaterials are materials with one or more of their dimensions in the order of 100 nm or less [6]. These materials usually exhibit unique and extraordinary physicochemical and biological properties compared to their bulk counterparts due to their specialized structural and functional components, internally or on the surface [7]. Although the opportunities and applications offered by nanomaterials are unrivaled, they also pose considerable risk to both human health and the environment [8].

The use of ENMs in various industries is rapidly increasing; consequently, an increasing concentration of these nanomaterials is reaching various environments, including soil [9]. These ENMs are released into the environment at various stages, such as during their synthesis, manufacturing of nano-based products, during usage, and finally after disposal of these products [10]. Among these stages, the first two stages pose a relatively lower risk of ENMs release into the soil, as the preparation and incorporation of ENMs into various products is typically performed under controlled conditions. However, there is a high risk of ENMs reaching the soil during the usage and disposal phases, which pose imminent threat to the environment [11]. For example, in the usage phase, damaged nano-based products degrade and release ENMs into the soil contributing to ENM pollution, whereas in the end-of-life stage, nano-based products are usually disposed in the landfills, where the process of their degradation accelerates, which ultimately releases ENMs into the soil due to leaching [12]. In addition, the rampant use of nano-based products in agriculture and environmental remediation processes such as nano-pesticides, nano-fertilizers, nano-based adsorbent materials, etc., also poses significant danger to the soil [13]. As in most cases, these products are directly applied to the soil and plants, significantly increasing the chances of the faster release of ENMs into the soil [14]. ENMs may also reach the soil through wastewater.

**Figure 1:**
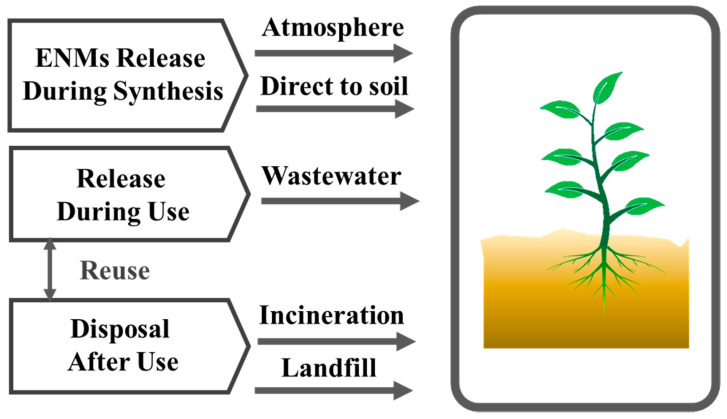
Possible ways of ENMs accumulation in soil.

Typically, during their lifecycle, many ENMs reach the soil, directly or indirectly, under various circumstances in significant quantities, making it essential to investigate the impact of these ENMs on soil ecosystem and the routes through which these ENMs enter the soil (cf. Figure 1) [15]. Apart from their release routes, the behavior of ENMs and their persistence in the soil also need to be studied. The role of nanomaterials in soil pollution can also be better understood by studying the ENMs life cycle in soil and how the soil properties influence their activity [16]. For instance, physicochemical properties of soil such as ionic composition, pH, temperature, hydrostatic pressure, etc., potentially affect the chemical form, mobility, bioavailability, and toxicity of ENMs [17].

Thus, it is crucial to study the effect of ENMs on the growth of the plant and on the microbiomes present in both plant and soil. Herein, we outline a basic introduction to nanomaterials and their synthesis and discuss in detail the effect of ENMs on the microbiomes present in plants and soil, which significantly affect the plant health and soil fertility [18,19]. ENMs seriously influence the interactions of microbiomes with the environment in different ways, which are discussed in detail. Moreover, some ENMs are microbicidal in nature and can selectively target a specific group of microorganisms, while others demonstrate growth promotion and beneficial effects [20]. The literature indicates an interesting trend whereby many ENMs demonstrate microbicidal activity at higher concentration, while at lower concentrations they promote the growth of microorganisms [20]. This review attempts to understand this difference in the activities of ENMs at different concentrations. In addition, the influence of ENMs on plant health, plant microbiome, and soil fertility is also briefly discussed.

## 2. Nanomaterials a Brief Introduction

One of the most diverse and popular classes of materials is nanomaterials, which are less than 100 nm in dimension and have a high specific surface area [21]. Due to their smaller size, ENMs can deeply penetrate into different living tissues, such as the blood–brain barrier and plant tissues [22]. The huge commercial applications of nanomaterials is due to the ability of fine-tuning which can significantly enhance their physicochemical properties [6]. These novel properties of nanomaterials have been exploited in a variety of industrial applications such as agriculture, cosmetics, aeronautics, pharmaceutics, etc. [6,23,24,25]. Several million tons of ENMs are discarded in different habitats including, landfills, sediments, soil, water, etc., both during preparation and after consumption [9,26]. Thus, proper choice of synthetic approach is essential for the design and synthesis of desired shapes and size of ENMs. Therefore, in the following section, we briefly discuss some common methods which are applied for nanoparticle synthesis. 

### 2.1. Approaches for Nanomaterial Synthesis

Usually, the selection of the method of ENMs synthesis is based on desirable properties of resultants nanomaterials including their size and other physicochemical characteristics [27,28,29,30]. Therefore, depending on the type of applications, the syntheses of nanomaterials are broadly classified into top-down approaches and *bottom-up* approaches, which are generally differentiated by the phase of the starting materials [31,32]. While, in the case of top-down methods, the reactants are often solids, in *bottom-up* methods, liquid or gaseous reactants are mostly used. 

### 2.2. Top-Down Approaches for the Synthesis of Nanoparticles

In the *top-down* methods, starting materials are processed in the solid-state, and therefore, these approaches are also called as physical processing methods [33]. In these approaches, miniaturization of large particles of bulk materials is performed to convert them into smaller sizes using a variety of physical methods including grinding, crushing, milling, etching, and other lithographic approaches [34]. The benefits of these approaches involve the scalable preparation of ENMs, and if required, the resulting ENMs can also be deposited over large substrates, such as metal oxides (silica, alumina, etc.) and layered and porous materials [35]. Since these processes do not involve the application of chemicals, tedious purification steps can be avoided. However, these methods are often not suitable for the controlled synthesis of ENMs. Additionally, due to there being less control over the surface properties of ENMs in these techniques, the preparation of the desired morphology of ENMs is often unattainable [36]. Therefore, herein we only discuss the *bottom-up* methods in more detail. Readers interested in scalable top-down methods for the fabrication of shape-specific ENMs may refer to the excellent reviews by Merkel et al. and Fu et al. [32,37].

### 2.3. Bottom-Up Approaches for the Synthesis of Nanoparticles 

In these methods, materials are fabricated using atoms, molecules, or clusters from the bottom [38,39,40]. The *bottom-up* approaches are broadly categorized in three different groups including physical methods, chemical methods, and biological methods [41]. The chemical methods are the most popular, since due to their efficiency in controlling the growth of particles, they are capable of fabricating complex nanostructures [42].

#### 2.3.1. Physical Methods for the Synthesis of Nanoparticles

Physical methods such as abrasion, condensation, melting, evaporation, etc., utilize mechanical pressure, high electrical or thermal energy, and radiations to produce a variety of ENMs [43,44,45]. Examples of top-down physical techniques are milling, crushing, etching, etc., which use solid state precursors [46], whereas the *bottom-up* physical methods such as chemical vapor deposition (CVD), flame pyrolysis, electrolysis, etc., apply gaseous or liquid materials as precursors [47,48,49].

#### 2.3.2. Chemical Methods for the Synthesis of Nanoparticles

Chemical methods are *bottom-up* techniques involving the application of chemical substances for the preparation of ENMs in the liquid phase [50]. In these processes, atoms or molecules are delivered to the nucleation sites which subsequently aggregate to form nanoparticles [51]. So far, a large number of chemical methods have been developed to produce high-quality, monodisperse ENMs by the proper control of the formation mechanism in solution [52]. Benefits of chemical methods include the formation of colloidal nanoparticles in the liquid phase, which can be easily separated in powder form; reactions are easy to perform which may produce a variety of different sizes and shapes of ENMs [53]. In the following sections, some of most common chemical methodologies applied for the synthesis of ENMs, such as the sol–gel method, microemulsion technique, hydrothermal synthesis, polyol synthesis, etc., are highlighted.

##### Sol–Gel Method

Sol–gel is a wet chemical technique for the synthesis of nanomaterials such as metal oxides, mixed metal oxides, etc. [54]. It has great potential for controlling the texture and surface properties of nanomaterials. This method involves various steps such as hydrolysis, condensation, and drying processes for the production of ENMs [51]. The colloidal suspension of particles in a liquid is called *‘sol’*, whereas *‘gel’* is a liquid containing polymeric materials [55,56,57]. This process is typically performed in water, but non-aqueous solvents are also commonly used depending upon the nature of the product [58,59]. This method has been used for the preparation of various metal and mixed metal oxides [59,60,61].

##### Microemulsion Method 

Microemulsions are homogeneous dispersions, which are optically transparent and thermally stable [62]. Surfactants are important in microemulsions, which function as interfacial layer and facilitate the separation of the aqueous and organic phases and prevent the agglomeration of droplets [63]. This interfacial layer forms different microstructures of spherical droplets such as water-in-oil (w/o) and/or oil-in-water (o/w) depending on the surfactant applied [64]. The former consists of oil droplets dispersed in a continuous aqueous phase over a bicontinuous “sponge” phase, whereas the later contains water droplets dispersed in a continuous oil phase. The water-in-oil microemulsions which are also known as reverse micellar systems act as excellent nanoreactors for the preparation of monodisperse ENMs [65]. This method offers several novel properties including large interfacial area, low interfacial tension, excellent thermodynamic stability and enhanced solubility of immiscible liquids [66]. This technique has been commonly used for the synthesis of a variety of metallic and metal oxides nanoparticles [67,68,69,70].

##### Hydrothermal Method

It is a solution reaction-based eco-friendly technique for the preparation of ENMs, which applies organic and/or aqueous solutions as a reaction medium in a closed vessel to achieve a high-temperature, high-pressure environment [71]. During this process, dissolution and recrystallization of chemical substances can be achieved which are poorly soluble or insoluble under normal conditions [72]. It is a single-pot process which produces highly crystalline nanomaterials which do not require any post-synthetic processing such as annealing, calcination, etc. [73,74]. The hydrothermal processes can be performed by a batch hydrothermal system or by a continuous hydrothermal process [75]. The former is a simple process which is able to control the oxidation states of the elements and can also prepare a system with a required ratio of phases containing a single element in different oxidation states, whereas using the continuous hydrothermal technique, high reaction rates can be achieved at a short time (<1 min) [76,77]. This technique can be potentially used for the large-scale synthesis of high-quality ENMs at low price [78,79,80,81].

##### Polyol Method

This is a versatile and simple technique, which is commonly used for the preparation of high-quality nanoparticles applying metal slats as precursors. These precursors are reduced at high temperatures using polyols as reducing agents [82,83]. Polyols are simple, low molecular weight glycols, such as ethylene glycol (EG) and its higher molecular weight counterparts like diethylene glycol (DEG), triethylene glycol (TrEG), polyethylene glycol (PEG), etc. [84]. Polyols not only act as reaction medium but also play the role of solvent, reducing agent and complexing ligands [85]. High dielectric constant of the polyols makes them good solvents for a variety of metal precursors. In addition, the high boiling points (up to 320 °C) and temperature-dependent reducing properties of polyols allow controlled nucleation and growth of particles to produce high-quality ENMs [86,87,88,89,90,91]. 

## 3. Plant and Soil Microbiome

Recent microbiome studies of various habitats have provided evidence that microorganisms are abundant and ubiquitous and play irreplaceable, diverse, and vital roles in nature [92,93]. Soil microbiome: Soil is a habitat which harbors the highest density of microorganisms, with up to 10^8^ bacterial cells per gram of soil, contributing significantly to the soil biomass [94]. The abundance and the diversity of microorganisms in the soil depend on the soil type, climatic conditions, edaphic factors, available nutrients, and oxygen availability [95]. As it is evident that these parameters vary greatly from place to place, the microbial communities may also differ. Interestingly, microbial communities in soil are reported to change at a very smaller distance, indeed, within a distance of a few millimeters [96,97,98,99]. 

Recently, the literature on soil microbiome has revealed that a majority of the archaeal and bacterial species present in different types of soils are unique, and only a few microorganisms are found in abundance among most of the samples analyzed [100]. It was also reported that the biomass of fungi and bacteria in the soil is 10^2^–10^4^ times higher than the biomass of other microorganisms including viruses, archaea, and protists [101,102]. The data of a variety of (66) soil samples presented in one such study shows the predominance of fungi belonging to the *Archaeorhizomycetes*, *Leotiomycetes,* *Agaricomycetes*, *Sordariomycetes*, *Dothideomycetes*, *Glomeromycota,* *Eurotiomycetes*, *Chytridiomycota,* and *Zygomycota* [100]. While bacteria predominantly included the members of Verrucomicrobia, Acidobacteria, Proteobacteria (Alphaproteobacteria, Betaproteobacteria, Gammaproteobacteria, and Deltaproteobacteria), Planctomycetes, Bacteroidetes, and Actinobacteria. Among Archaea, members of Crenarchaeota and Euryarchaeota were found predominantly in the studied samples [100]. These microorganisms in soil play vital roles such as nutrient solubilization (like zinc and phosphate solubilization), mineralization, and nutrient fixation [103,104]. Microorganisms mediated processes such as methane production and denitrification may also result in the loss of nutrients from the soil. Therefore, microorganisms are crucial in nutrient cycling [105]. 

Apart from soil microbiome, other organic matters of the soil such as humic substances (HS) have been known to play a vital role in plant growth, yield, and nutrition [106]. HS belong to naturally occurring organic substances which originate from the decomposition and transformation of plant, animal, and microbial residues [107,108]. The process of decay and transformation of plants and microbial remains in the soil generates a variety of highly acidic, relatively large, and colorful molecules which constitute HS. These substances enhance plant growth by increasing fertilizer efficiency, reducing soil compaction, and stimulating root growth by regulating metals concentration including iron and zinc [109,110]. Apart from this, various other physiological effects of HS on several aspects of plant growth and metabolism have also been reported [111]. 

Plant microbiome: Plant microbiome includes all the microorganisms that live on various parts of plants. These include microorganisms living on the aerial parts of the plants such as leaf (phyllosphere) and external surface of roots (rhizoplane), in a narrow region of soil that is directly influenced by root secretions (rhizosphere) and on the interior parts of the plants (endosphere), [112]. The interactions between the plant and its microbiome are complex and are influenced by numerous factors such as the plant type, age and health, secretions, surrounding environment, physicochemical conditions, initial microbial load in soil, etc. [113]. Interestingly, it has been confirmed that the specific microorganisms are selected by plants for the colonization of their rhizosphere [114]. Moreover, microorganisms present within a plant, i.e., in the endosphere, phyllosphere, and rhizosphere, may also change considerably even under an identical set of conditions [115]. Hence, most of the studies on plant microbiome have focused on (i) the relationship among various microorganisms including protists, archaea, bacteria, and fungi; (ii) the microbiomes that are plant-specific even at cultivar level; (iii) the vertical transmission of core microbiomes; (iv) the behavior of endophytes; and lastly, (v) the unpredicted functions and metabolic interactions [116]. There are various forms of interactions that take place between plant and their microbiomes. Some microorganisms cause diseases in the plants, while others are beneficial for the plants [117]. The plant associated microorganisms perform various life-support functions for the plants. Plants even employ specific microbial groups to perform specific functions by the discharge of specific plant exudates [118]. The traditionally known wide array of plant support functions offered by bacteria include nutrient fixation [119], nutrient mobilization [120], production of effector molecules [121], sequestration of micronutrients [122], stress tolerance, and protection against plant pathogens. Classical examples of microbes providing nutrients to host plant include Rhizobia, a diazotrophic bacteria, which forms root nodules in legumes and mycorrhizal fungi [123], while the nutrient mobilizing bacteria like phosphate solubilizing bacteria (e.g., *Pseudomonas* sp. and *Pantoea* sp.) are known to convert insoluble form of phosphate such as Ca_3_(PO_4_)_2_, lecithin, and powered phosphate rock into soluble form making it available to the plants [120]. Similarly, various molecules like IAA are produced by different microorganisms that are known to promote plant growth [124]. Micronutrients like zinc are also made available to the plants by bacteria through production of organic acids or Siderophores [122]. In addition to these traditionally known plant growth promoting activities, it has been recently demonstrated that microbes associated with plants play an even greater role in plant health and reproduction. Interestingly, it was observed that the biomass and flowering time of *Arabidopsis thaliana* is controlled by its characteristic microbiome [125]. In another study, it was found that when exposed to the disease, microbial communities in the rhizospheric region of *A*. *thaliana* play a vital role in its defense [118]. A similar protective role of the microbial community against wilt disease has also been demonstrated in the wild species of tobacco *Nicotiana attenuata* [126]. In a study on *Phaseolus vulgaris,* it was found that domestication affects the composition of plant microbiome [127]. Rhizosphere of the wild plant was found to possess a high population of Bacteroidetes. However, upon domestication, a decline in the population of the Bacteroides group and an increase in the populations of Actinobacteria and Proteobacteria was observed [128]. Furthermore, domestication adversely affected the population of symbiotic mycorrhiza and nitrogen fixers [129]. 

Comprehensive investigations of microbiomes present in the plants may assist in fostering sustainable agriculture by reducing reliance on pesticides and chemical fertilizers, while increasing the nutrient value of the crops and agricultural productivity [130]. Plants host different types of microbiomes that may change with varying conditions, which necessitates the understanding and investigation of the rather stable plant-specific core microbiome. Such microbiomes can be tailored for specific requirements such as better growth, defense against diseases, and for improving the quality of agricultural products [131]. Such extensive and systematic investigations will help in making agriculture more sustainable and less dependent on agrochemicals. Another important aspect will be to understand the harmful effects of pollutants of soil on the microbiomes of plant and soil [132]. Unmatched and rapid urbanization in countries like India and China has drastically enhanced soil pollution [133]. It is estimated that 16% of the soil in China including a vast area of farmland (19.4%) is contaminated, while most of these pollutants (~80%) are inorganic pollutants which are hazardous and highly toxic [134]. Among various inorganic pollutants, nanomaterials are emerging as one of the most widely used materials that may end up in the soil [135]. Reviews have estimated that several tons of these nanomaterials that are being annually produced finally end up in landfill and soil [9,136]. Owing to their known antimicrobial activities, it is obvious that these nanomaterials perturb the plant and soil microbiome homeostasis [137]. Hence, this review discusses in detail the soil pollution due to ENMs and their effect on the microbiomes of plants and soil.

## 4. ENMs in Soil, Their Release Routes, and Fate 

A variety of natural nanomaterials are present in the soil including clay, organic materials, and several metal and metal oxide nanoparticles [138]. However, several ENMs as discussed above are also discarded in soil and water due to the wide scale application of nanomaterials-based products [139]. According to various estimates, several million tons of a variety of metal and metal oxide nanoparticles including, Ag, Au, SiO_2_, ZnO, TiO_2_, and CNT (carbon nanotube) fullerenes are produced worldwide annually [140]. These ENMs are released in various environments intentionally or unintentionally, (a) during the preparation, (b) during their use, and (c) and while they are disposed of in the environment. Some of the products contributing significant quantities of ENMs to the environment include coating, pigments, paints, electronic, cosmetics, and optics [141,142]. Roughly 0.1 to 2% of the total ENMs produced reach the environment during the production phase [9]. On the other hand, during their usage, the amount of ENMs released into the environment is dependent on the type of the product used (Figure 1), while most of the nanomaterials used in electronics, paper, packaging, board, and plastics are released into soil and landfills [9]. It is also important to understand that ~60–90% of ENMs are disposed of in landfill, while the second highest quantity (~10–25%) is disposed of in the soil. This shows that tons of these ENMs are reaching the soil annually. SiO_2_, Titania (TiO_2_), zinc oxides (ZnO), iron, and alumina (Al_2_O_3_) are the most abundant ENMs that are released in various environments [143,144]. 

Furthermore, the dynamic multimedia fate and transport model (nanoFate) developed by Garner et al. has predicted the time-dependent accumulation of ENMs in various environments in considerable concentrations [145]. Unfortunately, concentration of certain highly produced ENMs such as TiO_2_, ZnO, etc., often even exceeds the minimum toxic threshold [145]. The quantity of ENMs is significantly higher in agricultural soil compared to other soils due to the direct application of biosolids, irrigation with ENMs containing wastewater, the use of nanomaterials-based pesticides and fertilizers, etc., whereas urban soils consist of relatively lower amounts of ENMs which are mainly contributed by products like paints and coatings used in buildings and other materials. Typically, the use of various ENMs decides their fate in different environments, such as TiO_2_ which is widely applied in pigments and reaches directly into the landfills [146]. Moreover, while burning ENMs based products in waste incineration facilities, the chances of ENMs reaching the soil also increases manifold. ENMs like TiO_2_ reach the soil when irrigated with sewage treatment effluents containing ENMs. According to a study, irrigation with wastewater, which has a high possibility of having ENMs in it, significantly enhances the nanomaterials contents in soil; for instance, ~90 µg of TiO_2_ has been detected per kilogram of soil annually [147]. It was also found that the soil treated with sludge contains the highest concentrations of ENMs. It was also calculated in the same study that ~0.3–1.3 µg/kg of TiO_2_ reached the soil in Europe every year, while ~0.05 and 0.09 µg/kg/Y of ZnO is discarded into the soil in the USA and Europe, respectively. Similarly, high quantities of TiO_2_ followed by ZnO are reaching various environments, including the soil [147].

The effect of ENMs in the soil is heavily dependent on the type of the soil and the properties of ENMs [148]. Depending upon their bioavailability, ENMs may have strong interactions with the charge particles present in the soil, can be soluble in water available in the soil, or can potentially be absorbed by various living organisms in the soil [149]. Apart from the soil, ENMs can also reach other environments including sediments and water bodies. Various physicochemical properties of ENMs, including size, morphology, chemical characteristics, and other surface properties play a crucial role in determining the fate of ENMs in the soil, since these characteristics significantly alter the electrical, optical, and catalytical behavior of the particles [150]. In addition, the surfaces of ENMs are typically functionalized with inorganic or organic ligands and other polymeric surfactants to enhance their colloidal stability, which also affects their interaction with soil particles, such as formation of colloidal solution in the water present in the soil, aggregation, etc. [151,152]. All the ENMs undergo aging, chemical transformation, aggregation, and disaggregation in soil [153]. The chemical transformation of the ENMs in soil may include adsorption, desorption, dissolution, and sulfidation, which may also alter the surface properties of these ENMs. The physico-chemical and biological transformations of ENMs in the environment determine their fate and bioavailability to the plants and to other microorganisms [154]. The physical transformation mainly involves agglomeration and deposition/sedimentation, as ENMs rarely exist as individual particles due to their high surface energy [155]. Once ENMs are released into the environment, they tend to undergo homo-agglomeration (interaction between ENMs) or hetero-agglomeration (ENMs interaction with foreign organic and inorganic particles) [156]. Usually, the hetero-agglomeration of ENMs with soil particles tends to dominate over homo-agglomeration due to the relatively small quantity of ENMs [157], whereas other physical transformations (deposition/sedimentation) occur in three different stages including an initial slow stage which occurs in parallel with process of agglomeration, a rapid phase, and another slow phase due to the small quantity of particles in suspension. The rate of sedimentation is often dependent on the process of agglomeration. Such high rate of agglomeration accelerates the process of the deposition of the suspended ENMs on a solid surface which strongly reduces their mobility and bioavailability in the environment [158]. On the other hand, during the chemical transformation of ENMs in soil, a variety of different compounds are formed due to the release of metal ions from ENMs dissolution, which are adsorbed on the surface of soil particles. For instance, soft metals, such as Ag, Cu, Zn, etc., usually form metal sulfides due to their high tendency to form complexes with sulfur containing substances including bio-macromolecules and other inorganic-sulfur sediments present in the soil [159]. Hence, the life of different nanomaterials in various environments including soil varies with the inherent properties of ENMs and with various environmental factors [138].

## 5. Role of Nanomaterial in Plant Health and Microbiome

Although, several ENMs based commercial products including fertilizers for improving plant growth and yield, pesticides for plant disease management, and advance sensors for monitoring plant health and soil quality are currently available in the market, the widespread application of ENMs in the field of agriculture is still in its infancy [160,161,162]. ENMs based nanofertilizers are known to enhance the nutrients use efficiency, while the nanopesticides possess high efficacy, increased solubility, and durability with minimum amount of active ingredients [161,163,164]. In addition, several other benefits of ENMs based agricultural products have been extensively reported [165,166,167]. Therefore, the increasing use of ENMs in agriculture is also significantly increasing the risk of soil contamination with ENMs [162]. Indeed, the presence of large amounts of ENMs in the soil may considerably affect the plant rhizosphere due to the enhanced interactions of ENMs with soil and plant microbiome [168]. Particularly, when the ENMs are released into the subsurface to achieve short-term benefits, they can be potentially accumulated by the plants and may have negative impacts over longer periods of time [169]. Plants secrete various exudates supporting microbial growth in their rhizosphere, while microbes facilitate various nutrient cycles so as to support plant growth acting synergistically with plant roots [170]. However in the presence of ENMs, the microbial communities in the rhizosphere, exudates from the plants, and extracellular substances produced by the microbes are all significantly influenced [171]. Therefore, the presence of ENMs in the soil considerably affects the health of the plants either directly or through influencing the plant and soil microbiome. Such effects are discussed below in detail.

### 5.1. Direct Effect of ENMs on Plants

The uptake of ENMs by plants and higher living organisms and the subsequent interaction with various biomolecules is well-established [172,173]. Inside the soil, the interaction of ENMs with plants potentially affects plant physiology and possibly food security, due to nano-phytotoxicity which has been intensively studied [174,175]. Apart from the harmful effects, the beneficial effects on ENMs-plants interactions at the biochemical, physiological, and genetic levels are also reported by various authors (Table 1) [176,177,178]. These effects usually depend on various factors such as the chemical characteristic of ENMs, surface charge, their size, and doses [179,180,181,182,183]. In addition, the ability of ENMs to move within the soil, from soil to plants and to different tissues within the plant also plays an important role in determining the effect of ENMs on plant growth [184]. Therefore, proper understanding of the movement of ENMs within the soil and inside the plant is crucial to predict the actual impact of ENMs on plant growth [185]. The movement of ENMs depends on the physicochemical properties of both ENMs and the surrounding environment. The ENMs present in soil around the roots may be taken up by the roots and transported to the aerial parts of the plants including leaves and other aerial parts, directly influencing their growth [174]. Several studies have also been reported on whether these nanomaterials reach leaves and tissues of other aerial parts of the plant after being absorbed by the roots [186]. The ENMs have to cross a number of barriers to enter into plant tissues such as the cuticle and the plant cell wall. They can enter the plant tissues through injuries or through wall pores. It is demonstrated that some ENMs may result in the enlargement of cell wall pores to enter into the plant tissues even if the size of ENMs is larger than the pores in the wall [187]. CuO was shown to reduce cell wall oxyglucan and pectin content in *Arabidopsis thaliana* roots [188]. In addition, the aerial parts are directly exposed to the ENMs when ENMs are applied through foliar spray [189]. The foliar spray of ENMs facilitates the uptake of ENMs either through cuticular pathway or via stomatal openings. The cuticular pathway includes the transport of nonpolar solutes via diffusion and permeation and polar solutes through polar aqueous pores [190]. Transport of ENMs occurs through the vascular system after entering the leaf apoplast through the stomatal pathway [191]. For example, Wang et al. demonstrated the foliar uptake of ENMs through an aerosol process by the watermelon plant. The study divulged that ENMs entered the leaf through the stomatal pathway, passed through the stem, and reached the root of the plants [192]. The movement of nanomaterials in plants can be apoplastic or symplastic, i.e., either through extracellular spaces and xylem vessels or through the plasmodesmata, respectively [193]. For example, when the movement of TiO_2_ NPs and MWNCTs (multi-walled carbon nanotubes) from the soil into plants (wheat and red clover) was checked, Both TiO_2_ NPs and MWNCTs exhibited limited mobility from soil to leachates [194]. As far as the molecular mechanism of nanomaterial toxicity is concerned, various mechanisms have been proposed [195,196]. 

One of the most common consequences reported in the literature is the increased antioxidant activity. Which is measured in terms of increased activity of peroxide dismutase and superoxide dismutase. Upon addition of carbonaceous materials including reduced graphene oxide (RGO), MWCNTs, and fullerene to the rhizosphere of rice for a period of 30 days, the induction of four phytohormones including gibberellin, auxin, brassinosteroid and indoleacetic acid was observed. In addition to hormones, enhanced activities of peroxide dismutase and superoxide dismutase was also observed [197]. However, in the same study, CNMs led toxicity to rice plants and microbial communities has also been observed. In another study, 75 and 60% growth inhibition of zucchini (*Cucurbita pepo*) was observed in the presence of MWCNTs and Ag NPs, respectively [198]. Similarly, Ag NPs were found to inhibit the germination of ryegrass and flax (*Linum usitatissimum*) [199], and a higher concentration of Ag NPs minimized the germination of barley (*Hordeum vulgare* L.) [177]. In addition, another study revealed that CeO_2_ is accumulated in the roots which were exposed to soybean, and their accumulation and translocation in edible tissue was also reported [208]. When the plant was exposed to ZnO NPs up to a particular dose (200–300 mg/L), a decrease in its chlorophyll content was observed, which significantly affected plant health [200]. Further, the RGO was found to inhibit the process of photosynthesis in pea plants, which had serious effect on the biomass and carbon fixation [209]. 

In contrast, the plant growth-promoting activity of ENMs has also been reported [180,210]. Particularly, with lower doses, ENMs accelerate the biosynthesis of secondary metabolites, promote the activities of antioxidant enzymes, help to enhance water and fertilizer absorption efficiency, facilitate the process of photosynthesis, etc., which ultimately results in the stimulation of plant growth [211]. For example, TiO_2_ nanoparticles promoted plant growth consequently alleviating cadmium stress in soybean. The nanoparticles promoted plant growth through improving chlorophyll or carotene content which enhanced the rate of photosynthesis [201]. Apart from inorganic ENMs, other carbonaceous nanomaterials, including Carbon nanotubes (CNTs), graphene, etc., also possess excellent ability to penetrate the plant system and influence metabolic functions of the plants at appropriate concentrations [212]. In one study, graphene oxide promoted seed germination through increased water retention [213]. The treatment of aged rice with a small quantity of Ag NPs (5 to 10 ppm) enhanced seed germination and the seedling vigor [202]. Similarly, ZnO improved the tomato plants growth probably by serving as a micronutrient [180]. The treatment of gram (*Cicer arietinum*) plants with nano-onions (wsCNOs), which is a water-soluble wood-based pyrolysis waste product, enhanced the overall growth rate of the plant [205]. Some of these ENMs act as micronutrients for a variety of plants, but they can also serve as the carrier for other nutrients [211]. Due to their smaller size and exceptional penetration capabilities, ENMs are better carriers of nutrients [193]. Nano-cerium was found to increase the plant biomass when tested on *Arabidopsis thaliana* [214]. These observations were supported by the data of microarray published in the same study. Poly(acrylic acid) nano-ceria was also found to increase the carbon assimilation rates by as much as 67% in *A*. *thaliana* plants [215]. Nano-ceria was found to scavenge the free radicals protecting the plant against abiotic stress. This finding contradicts other studies where ENMs have been shown to actually induce oxidative stress in plants through ROS generation. When Tobacco cells were tested for the change in gene expression following exposure to carbon nanotube, it was found that lower exposure concentrations actually promoted cell growth. Genes involved in transport of water and cell division were upregulated [216]. When the effect of nano Fe_3_O_4_ on *Triticum aestivum* L. plants was evaluated, an increase in the antioxidant enzyme activity both in root and aerial parts was observed promoting the overall growth of the plant [217]. Despite of the vast literature, further systematic studies especially on the mechanism underlying the growth promotion are required. The harmful effect of an environmental pollutant a DDT metabolite (dichloro diphenyl dichloroethylene; p,p′-DDE) on the plants became significant in the proximity of fullerene NPs [218]. This study has demonstrated that the presence of ENMs in soil may also influence the uptake and toxicity of other environmental pollutants. Careful studies therefore should be carried out at realistic doses to reach any conclusion regarding the toxicity or growth promoting activity of these ENMs.

### 5.2. Influence of ENMs on Plant Microbiome and Soil Microbiome

**Figure 2:**
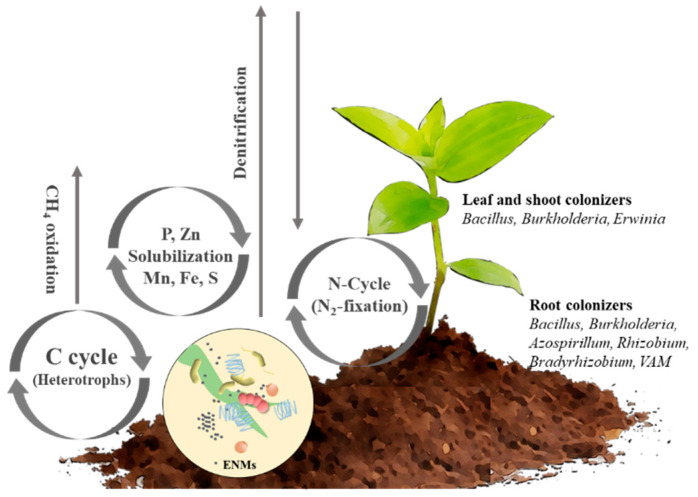
Versatile roles played by microorganisms in the geochemical cycling of nutrients.

ENMs may affect the plant growth directly or indirectly by influencing the microbial community of the soil or the plant (Figure 2, Table 1). For instance, it has been observed that Nano TiO_2_ and ZnO influence the microbial communities in the soil, and the toxicity of ZnO was more pronounced than that of TiO_2_ [219]. Population of nitrogen fixing and methane oxidizing bacteria decreased significantly following the treatment with ENMs, while the population of well-known decomposers of recalcitrant organic pollutants, i.e., the members of the family Sphingomonadaceae, increased significantly. When the effect of TiO_2_, one of the most abundant soil ENMs, was checked on the wheat microbiome, it was found that although the populations of certain groups of microorganisms changed root colonization by arbuscular mycorrhiza while plant growth remained largely unaffected [220]. In addition, the change in microbial community can serve as a marker for the contamination of soil by TiO_2_ NPs. Furthermore, another study evaluated in detail the impact of Ag NPs on soil microbial communities, and it was found that Ag NPs significantly affected microbial communities in the soil [221]. The same group demonstrated earlier that the populations of ammonia oxidizers and β-proteobacteria decreased significantly following exposure to Ag NPs, while the populations of Acidobacteria, Actinobacteria, and Bacteroidetes increased significantly [204]. Ag NPs exposure also led to a decrease in the abundance of nitrogen-fixers, soil microbial biomass, and the leucine aminopeptidase activity [204]. When the soils were treated with C60 fullerenes with an average size of 50 nm, a three- to four-fold decrease in the population of fast-growing bacteria was observed [222]. In yet another study, the addition of TiO_2_ and polystyrene ENMs in the rhizosphere of lettuce seedling decreased the population of rhizospheric bacteria, consequently inhibiting the shoot and root growth [168]. When the soils were irrigated with wastewater containing ENMs, an increase in the population of cyanobacteria and a variety of unknown Archaea were observed, together with a significant reduction of the life cycle of *A*. *thaliana* [223]. Carbon nanomaterials also resulted in a change of the microbial community of rice rhizosphere and incurred environmental toxicity [197]. In a study of tomato plants, the treatment of soil with CNTs did not change the microbial community of the soil significantly [224]. Among various carbon nanomaterial tested, the maximum change of the microbial community was observed with reduced graphene oxide. Doolette et al. investigated the toxicity of sulfidised-silver nanoparticles (Ag_2_S-NPs) on soil microbial communities, using a combination of 16S rRNA amplicon sequencing, quantitative PCR, and species sensitivity distribution methods. These approaches helped in calculating the toxicity thresholds (HCx, hazardous concentrations) of Ag NPs to the microbial communities in the soil. At the HC20 (80% of species protected), soil operational taxonomic units were less sensitive to Ag_2_S-NPs than AgNPs and Ag^+^ (5.9, 1.4 and 1.4 mg Ag kg^−1^, respectively) [225]. 

The mechanism underlying the well-known antimicrobial activity of ENMs has also been studied and reported extensively [226,227]. Among several mechanisms, some of the common mechanisms involve metabolism perturbation (purine metabolism), cell membrane disruption, DNA damage, protein denaturation, free radical formation and induction of oxidative stress, inhibition of respiration, mutagenesis, etc. [137]. The actions of ENMs may not be always microbicidal, but in some cases, they can be inhibitory to various enzymes and metabolic processes [210]. Often, the toxicity of ENMs is based on a variety of physicochemical properties of particles including, size, morphology, chemical characteristics, hydrophobicity, etc. Moreover, some of the organisms are differentially sensitive to various nanomaterials [228]. For instance, some ENMs are more potent towards Gram +ve bacteria than the Gram -ve bacteria [229]. The rate of bacterial growth and the capability of bacteria to produce extracellular polysaccharides also influence the sensitivity of bacteria to the ENMs [230].

## 6. Effect of ENMs on Microbes Mediated Processes and Nutrient Cycle

ENMs gradually accumulate in the soil through different routes, and many of these are microbicidal in nature at least at higher doses. Hence, these ENMs can potentially inhibit the key microbial biogeochemical processes [215]. These processes include ammonification, denitrification, nitrogen fixation, phosphate solubilization, and plant growth promoting (PGP) activities, crucial for maintaining the soil fertility and ecosystem (Figure 2) [231]. For example, in such a study, McGee et al. have evaluated the toxicity of Ag NPs to bacterial and fungal communities in an agricultural pasture land soil using microcosm-based experiments [232], wherein the combination of enzyme analysis, molecular fingerprinting, and amplicon sequencing was used [232]. It was observed that the studied AgNPs were toxic to a variety of microorganisms in the soil, considerably reducing the total soil dehydrogenase and urease activity. Substantial shifts in bacterial community composition were also observed which were characterized by a drastic decrease in the population of Acidobacteria and Verrucomicrobia and an increase in the population of Proteobacteria. The same group also investigated the effect of various concentrations of microsized Ag particles on the bacterial and fungal community structures of an agricultural pastureland soil using similar approaches [233]. The study revealed a significant impact of Ag contamination on the soil enzyme processes leading to a reduction of soil dehydrogenase activity. Furthermore, the copy numbers of the *amo*A gene also decreased considerably with the presence of AgNPs, demonstrating higher sensitivity to archaeal ammonia oxidizers’ than bacteria. Apart from this, various reports also exist on the effect of other ENMs on various microorganisms which are involved in these geochemical processes [210,234]. An extensive literature survey reveals that the toxicity of ENMs is typically measured during the test at high concentrations of ENMs. Contrarily, some ENMs at lower concentrations have been known to promote certain biogeochemical processes, which ultimately promote plant growth as discussed below [20,235]. 

Thus far, several studies have been reported on the effect of ENMs on microbes mediated cycling of a variety of crucial nutrients such as carbon and nitrogen [236]. Ag NP-treated soil samples exhibit reduced activities of acid phosphatase, β-glucosaminidase, β-glucosidase, and arylsulfatase, which are essential enzymes for the carbon and other nutrient cycling [237]. Nano-sized CuO were found to inhibit the three important soil processes, namely, nitrification, denitrification, and soil respiration. However, the inhibition was noticed at a very high concentration (100 mg/kg). Although microorganisms play an irreplaceable role in various steps of the nitrogen cycle including ammonification, denitrification, and nitrogen fixation. Among these, the nitrogen fixation is a most crucial process, which is performed by both symbiotic and free-living microorganisms. The effect of various ENMs on the nitrogen fixation activity of both symbiotic and free-living microorganisms has been widely reported. It was found that in the presence of TiO_2_ NPs, the growth rate of *Anabaena variabilis*, its nitrogen fixation rate, and the rates of nitrogen storage declined [238]. One matter of concern reported in the study is that the time of exposure is a more important factor than the concentration of ENMs. MWNCT at a concentration of 100 µg/kg of soil increased the nitrogen fixation activity, resulting in an increase of the plant biomass [235]. The negative effect of copper ENMs on the microbial carbon and nitrogen cycle is also reported, which is not found to be inhibitory at lower concentrations (0.1–1 mg/kg of soil) [239]. Furthermore, denitrification was the most sensitive microbial process to CuO-NPs [206]. In another study, a high concentration of nano-CeO_2_ inhibited nitrogen fixation in leguminous soybean crops [207]. The presence of several ENMs in the soil, such as Ag, Zn, and Ti, considerably decreased the nodulation frequency of *Medicago truncatula* by *Sinorhizobium meliloti* [240]. Among various ENMs, Ag has been widely studied due to its established antimicrobial properties. For example, in one study, AgNPs were reported to inhibit the growth of *Azotobacter vinelandii*, which is a free-living nitrogen fixer [241]. In most of the cases, the toxicity of ENMs has been largely dependent on size of the nanoparticles. Often, the ENMs caused oxidative stress, cell damage, inhibition of nitrogenase activity, and death by apoptosis. The influence of SWCNTs, MWCNTs, and GO (graphene oxide) on legume–Rhizobium symbiosis has also been studied [235]. At a low MWCNT concentrations (100 μg/mL), enhanced nitrogen fixation activity of nodules has been observed which increased the biomass by ~15-25%.

Reports on the effect of ENMs on various other processes and PGP activities such as nitrification, denitrification, potassium and phosphate solubilization, and microbial protection of plants against several diseases are also available. The Ag NPs were found to release silver ions inhibiting the nitrification activity of *Nitrosomonas europaea* [242]. In another study, silver NPs with a size of 20 nm compromised the outer membrane of the bacterium, inhibiting ammonium oxidase activity. Various concentrations of CeO_2_, SiO_2_, TiO_2_, and ZnO NPs were tested for their activity against plant growth promoting bacteria including *Azotobacter*, potassium and phosphate solubilizing bacteria, and their enzymatic activities [243]. At the tested concentration (1 mg/g), CeO_2_ and ZnO reduced metabolism and inhibited activity of various enzymes such as urease and catalase. These ENMs also reduced the population of *Azotobacter*, P-solubilizing, and K-solubilizing bacteria as evident from colony counts. The activity of alumina (Al_2_O_3_) and Silica (SiO_2_) NPs against plant growth promoting rhizobacteria and other bacteria, such as *Pseudomonas fluorescens*, *Bacillus brevis*, and *Bacillus megaterium*, was checked. The Al_2_O_3_ NPs were found to be significantly toxic to these test organisms at the high concentration of 1000 mg/L [244]. 

## 7. Activity of ENMs against Plant Pathogens

ENMs are also known to influence plant-pathogen interaction (cf. Figure 2). Nano-formulations of some pesticides are already in use, such as TiO_2_/Ag composite which is modified with sodium dodecyl sulfate and dimethomorph [245], but here, we limit our discussion to metal and metal oxide ENMs excluding the specially designed nanoformulation of conventional pesticides. Many studies have reported the anti- phytopathogenic activity of ENMs both in vitro and in vivo [246,247,248]. This is attributed to the nanoforms of the ENMs or to the metal ions released from these ENMs [20,196,249]. ENMs may also promote and augment the activity of microorganisms capable of inhibiting these phytopathogens. Several studies have been conducted to study the effect of silver on a variety of phytopathogens due to the importance of silver as antimicrobial agent. For instance, the inhibition properties of Ag NPs were tested on phytopathogenic fungi, which is known to cause a disease in ryegrass [248]. Similarly, the inhibition properties of several ENMs, such as MWCNTs, Fe_2_O_3_, etc., against Turnip mosaic virus and Tobacco mosaic virus were also investigated [197]. The ENMs significantly decreased the coat proteins of the viruses (15–60%), reducing their pathogenicity. However, the dose used for the foliar spray, from the viewpoint of the author, was quite high (50–200 mg/L) [197]. When low doses (500 ng/mL) of ENMs (Ag, SiO_2_, TiO_2_, and ZnO) were used to assess their effect on the antifungal activity of *Pseudomonas protegens* CHA0 against *Candida albicans,* it was found that the ENMs promoted the antifungal activity of *P*. *protegens* CHA0 [20]. The inhibition of *Fusarium graminearum,* a plant pathogen by ZnO NPs, has been reported both in vitro and in vivo [250]. Upon treating wheat plants with ZnO NPs, the chances of infection by *F*. *graminearum* are reduced significantly. A substantial decrease in *F*. *graminearum* colony forming unit was observed compared to control [251]. The treatment with Zn nanomaterials did not cause any damage to the plant, and the amount of zinc in the grains also remained within permissible limits. Apart from this, several carbonaceous materials, including RGO, activated carbon, C60, MWCNTs, etc., were evaluated against two phytopathogenic fungi (*F*. *graminearum* and *F*. *poae*) [252]. It was found that all the ENMs inhibited the growth of the two tested fungi except for C_60_ and AC. This inhibition was mainly due to the inactivation of the spores caused by (i) plasmolysis, (ii) spores blockage due to the accumulation of ENMs on spores surfaces, and (iii) obstruction in water uptake. Even in the field trials, Ag NPs were found to inhibit *Colletotrichum* spp., a phytopathogen causing anthracnose [253]. It was demonstrated that the use of nanoparticles before the onset of the disease considerably minimized the infection of *Colletotrichum* spp. in the pepper plant. It is therefore evident from the published literature that ENMs have great potential in protecting different types of plants against several infections by pathogens; however, their toxicity at higher concentration needs to be evaluated carefully, which still remained a serious concern.

## 8. Conclusions

Nanomaterials are nanosized forms of various organic and inorganic compounds with superior desirable properties. These nanomaterials can be engineered or tailored using chemical, physical, or biological methods for their use in various industries and sectors such as agriculture. Since these ENMs are being produced at the industrial scale, they are also being released in various environments including soil both intentionally and unintentionally, without evaluation of the risk. Among various materials, some of the ENMs being discarded into the soil in considerably large quantities include silver, SiO_2_, titania, iron, iron oxides, zinc oxide, alumina, etc. The continuous and unregulated release of tons of these ENMs has resulted in their accumulation in the soil. Studies are emerging not only on their accumulation but also on the change in their concentrations/kg of soil/year. This variation in concentration depends on the life span of ENMs in the soil and their release pattern. Upon reaching the soil, ENMs may have variable fates and life cycles in the soil. These ENMs are known to influence and interact with soil, plants, and plant and soil microbiomes. These interactions strongly influence the soil microbiome and plant microbiome, consequently affecting plant health. The activity of these ENMs depends on a number of factors such as size, shape, chemical nature, surface charge, hydrophobicity, etc. Notably, the interactions of nanomaterials with the soil ecosystem cannot be generalized as soil, soil microbiome, and plant microbiome vary from plant to plant and soil to soil. To date, several studies have investigated the effects of ENMs on various living forms in the soil, including microorganisms and plants. Microorganisms play very important roles in the biogeochemical cycling of nutrients and help in the mobilization and fixation of nutrients for plants, subsequently affecting the overall soil fertility and plant health. Unfortunately, many of these investigations have used very high doses of ENMs, while the realistic concentrations present in soil are far lower. Interestingly, in many studies, almost no effect was observed at lower doses. Some studies have even shown growth promoting and positive effects of low doses of ENMs, and hence, the use of realistic doses must be considered for realistic evaluation of their toxicity or growth promoting activity. Thus, the regulation of the release of ENMs during manufacturing, applications, and disposal after use is necessary. Similarly, studies focusing on growth promoting activities of ENMs have been seldom investigated and reported. Therefore, proper assessment of ENMs accumulation in the environment, particularly in the soil and plants, and their threat to the microbiomes is highly desirable. For example, although studies have demonstrated the plant growth promoting activity of Ag, its accumulation in plants and consumption of such crops by humans may be associated with elevated health risks, as their continuous and increasing release may pollute the soil beyond a limit with severe consequences. Therefore, the wise and sustainable use of nanotechnology in agriculture must be ensured by carefully evaluating all the parameters and associated risks. In this regard, biocompatible ENMs are a suitable alternative, which being biodegradable, remain in the soil for a shorter time. In addition, these materials are less harmful to the soil microbiomes and are easily recyclable. To conclude, ENMs are a boon for the soil if their concentration in it is very low and are regulated and crafted according to the needs. However, unregulated release of ENMs in the environment potentially enhances their concentration in the soil which may cause a serious threat to food security and crop productivity, also leading to health hazards for the human population and permanently damaging the status of the soil as a valuable resource. 

## Figures and Tables

**Table 1:** Various consequences of the nanomaterial interactions with the plant, plant microbiome, and the plant growth promoting activities of microorganisms.

S.No.	Nanomaterial	Tested Organism	Dose	Outcome	Reference
**Direct Plan-Nanomaterial interaction**					
*Inhibitory Effect*					
1	MWNCTs	Rice	50–500 mg/kg soil	Phytohormone induction	[197]
2	Ag & MWNCT	Zucchini	100–500 mg/L	Growth Inhibition	[198]
3	Ag	Ryegrass and Flax	40 mg/L	Decreased germination	[199]
4	Ag	Barley	1.5 g/L	Decreased germination	[177]
5	ZnO	*Arabidopsis thaliana*	200–300 mg/L	Decreased Chlorophyll Content	[200]
*Growth Promoting effect*					
					
6	TiO_2_	Soybean	100–300 mg/kg soil	Enhanced Photosynthesis	[201]
7	Ag	Rice	5 and 10 ppm	Enhanced Seed Germination	[202]
8	ZnO	Tomato	8 mg/L	Enhanced Photosynthesis	[180]
9	Ce	*Arabidopsis thaliana*	500 mg/L	Increased C assimilation	[203]
**Effect on Plant microbiome and Microbes mediated Biogeochemical cycling**					
10	Ag	N_2_ fixers and Methane oxidizers	1 mg/kg soil	Decreased population	[204]
11	TiO_2_	Rhizospheric bacteria	100 µg/ml	Decreased Growth	[168]
12	MWCNTs	N_2_-fixers	100 µg/kg Soil	Increased N_2_-fixation and Plant biomass	[205]
13	CuO	Denitrifiers and Nitrifiers	500 mg/kg	Inhibition	[206]
14	CeO_2_	N_2_-fixers	1 g/kg soil	Inhibition	[207]

## Data Availability

Data contained within the article.
